# Patients views on a new surveillance pathway involving allied non-medical staff for people with treated diabetic macular oedema and proliferative diabetic retinopathy

**DOI:** 10.1038/s41433-022-02050-1

**Published:** 2022-05-06

**Authors:** Lindsay Prior, Noemi Lois, on behalf of the EMERALD Study Group, Ahmed Saad, Augusto Azuara-Blanco, Caroline Styles, Clare Bailey, Danny McAuley, David H. Steel, Faruque D. Ghanchi, Geeta Menon, Haralabos Eleftheriadis, Stefanos Efraimidis, Jonathan Cook, Ariel Wang, William Sones, Nachiketa Acharya, Noemi Lois, Norman Waugh, Hema Mistry, Mandy Maredza, Samia Fatum, Sobha Sivaprasad, Stephen Aldington, Peter H. Scanlon, Katerina Ivanova, Tariq M. Aslam, Victor Chong, Andrew Jackson, Christine McNally, Rachael Rice, Lindsay Prior

**Affiliations:** 1 Centre for Public Health, Queen’s University Belfast, Belfast, Northern Island UK; 2 The Wellcome-Wolfson Institute for Experimental Medicine, Queen’s University, Belfast, Northern Ireland UK; 3 The Belfast Health and Social Care Trust, Belfast, Northern Ireland UK; 4 South Tees Hospitals NHS Foundation Trust, Middlesbrough, England UK; 5 Queen’s Margaret Hospital, Fife, Scotland UK; 6 University Hospitals Bristol NHS Foundation, Bristol, England UK; 7 Sunderland Eye Infirmary, City Hospitals Sunderland NHS Foundation Trust, Sunderland, England UK; 8 Bradford Teaching Hospitals NHS Trust, Leeds Bradford, England UK; 9 Frimley Park Hospital NHS Foundation Trust, Surrey, England UK; 10 Kings College Hospital NHS Foundation Trust, London, England UK; 11 Centre for Statistics in Medicine, University of Oxford, Oxford, England UK; 12 Sheffield Teaching Hospitals NHS Foundation Trust, Sheffield, England UK; 13 Warwick University, Warwick, England UK; 14 John Radcliffe Hospital, Oxford University Hospitals NHS Foundation Trust, Oxford, England UK; 15 Moorfields Eye Hospital NHS Foundation Trust, London, England UK; 16 Gloucestershire Hospitals NHS Foundation Trust, Gloucester, England UK; 17 Manchester Royal Eye Hospital, Central Manchester University Hospitals NHS Foundation Trust, Manchester, England UK; 18 Royal Free Hospital NHS Foundation Trust, London, London, England UK; 19 Northern Ireland Clinical Trials Unit, Belfast Health and Social Care Trust, Belfast, Northern Ireland UK

**Keywords:** Health care, Retinal diseases

## Abstract

**Background/Objective:**

To explore acceptability by patients and health care professionals of a new surveillance pathway for people with previously treated and stable diabetic macular oedema (DMO) and/or proliferative diabetic retinopathy (PDR).

**Subject/Methods:**

Structured discussions in 10 focus groups with patients; two with ophthalmic photographers/graders, and one with ophthalmologists, held across the UK as part of a large diagnostic accuracy study (EMERALD).

**Results:**

The most prominent issues raised by patients concerned (i) expertise of the various professionals within clinic, (ii) quality of interactions with clinic professionals, especially the flow of information from professionals to patients, and (iii) wish to be treated holistically. Ophthalmologists suggested such issues could be best dealt with via a programme of patient education and tended to overlook deeper implications of patient concerns for the organisation of services.

**Conclusion:**

For patients, the clinical service should not only include the identification and treatment of disease but also exchange of information, reassurance, and mitigation of anxiety. Alterations in the standard care pathway need to take account of such concerns and their implications, in addition to any assessments of ‘efficiency’ that may flow from changes in diagnostic technology, or the division of professional labour.

## Introduction

Ophthalmology is often the busiest outpatient speciality in Hospitals, accounting for 8% of all outpatient activity [1]. Considering the high number of people with diabetes (in the United Kingdom over 4 million, which equates to a 6% of the population) [2] and given the increasing prevalence of diabetes, diabetic retinopathy (DR) and its sight threatening complications, diabetic macular oedema (DMO) and proliferative diabetic retinopathy (PDR), ophthalmology is likely to experience a rise in the number of outpatient visits related to this disease. Problems are accentuated further by the requirement for patients with DMO and/or PDR to return to clinics at short intervals to receive treatment until their condition has been controlled and, thereafter, for life. Discrepancies between capacity and demand exist, and it is known that delayed appointments may lead to poorer visual outcomes for patients [3].

One response to a growing demand for scarce medical resources is to re-organise services. With this in mind, Effectiveness of Multimodal imaging for the Evaluation of Retinal oedema And new vesseLs in Diabetic retinopathy (EMERALD) sought to determine whether patients with DMO and/or PDR previously successfully treated (i.e. DMO cleared and PDR became quiescent) could be followed by multimodal retinal imaging and review of these images by trained ophthalmic graders [4]. EMERALD demonstrated the new ophthalmic grader pathway had adequate sensitivity to detect active disease (DMO and PDR), albeit with lower specificity [5]. This new form of surveillance would be well suited to a re-design of ophthalmic services including provision of ophthalmic grader-led “live” and “virtual” clinics. In “live clinics” images are obtained, evaluated, and results communicated to patients all on the same day. In “virtual clinics” evaluation of images does not occur on the same day; results are subsequently communicated to patients remotely (e.g. by post). Virtual clinics are increasingly used in ophthalmology; to date, these are predominantly undertaken by ophthalmologists [6–11]. There is none or scarce information on the acceptability of grader’s-led and virtual clinics, respectively, to patients and health professionals [10, 12]. EMERALD explored this.

## Methods

To determine the acceptability of the new ophthalmic grader pathway to patients and health care professionals, focus groups (FGs) were organised in 5 of the 13 sites in the United Kingdom participating in EMERALD. Patients eligible and willing to participate in EMERALD were invited to attend FG discussions; informed consent was obtained for all participants. A total of 36 patients with either DMO, and/or PDR, took part in FG discussions.

Activities within the FGs were designed to follow a common format; proceedings were sufficiently flexible to allow participants to talk about key issues in their own terms, and to add their own concerns to those of the moderator [13]. The first stage of the meetings gathered material on patient views about various features of a standard clinic: waiting time in clinic, interactions with nurses during visual acuity testing; interactions with photographers, and time with the ophthalmologist. The prompts used for such discussions were a series of still photographs that reflected each phase of the standard care pathway (e.g. photograph of a patient undergoing a slit-lamp biomicroscopic examination by an ophthalmologist).

The second stage of the group discussions gathered reactions to the provision of a virtual clinic. Such provision was explored via the use of three fictional scenarios or vignettes [14, 15]. Vignettes offered brief stories of imaginary patients who attended a clinic in which images of the retina were taken, were told that they would be informed of the results of their visit (by letter) on another day, and that no meeting with the ophthalmologist would take place ‘today’. The words ‘virtual clinic’ were not used by either moderator or participants. During discussions, participants voiced a series of issues unanticipated or outside the planned script (one of the benefits of using FGs). Unanticipated topics included the potential use of artificial intelligence for diagnosing eye conditions, the need for a holistic approach to the treatment and management of diabetes, and concerns about anxiety. Views and opinions relating to matters of trust and confidence (in health professionals) and expertise of clinic personnel were threaded throughout the discussions.

Following the patient FGs, three further meetings were conducted; two with ophthalmic photographers and graders from four of the 13 study sites (*n* = 7 individuals) and one FG with ophthalmologists (*n* = 6) from 6 participating sites. The ‘focus’ for these three meetings was on views and opinions about care pathways derived from the patient group discussions.

**Fig. 1:**
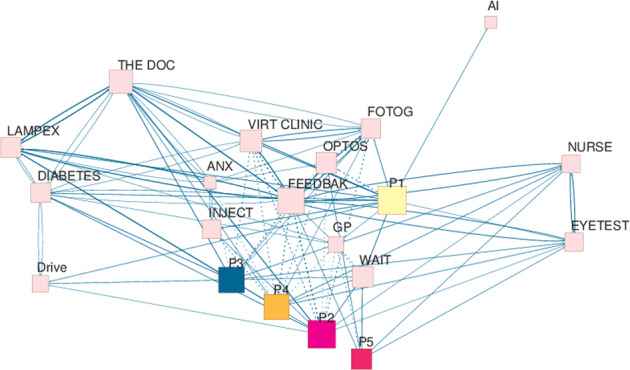
Issues discussed in focus group (FG) Discussion 5. The web was constructed using textual codes and numerical counts. Initially, each ‘turn’ (or phase of talk) in the FG transcript was linked to an identifiable speaker. Following that, the content of the turn was allocated to a node label or code (sometimes a number of codes). Node labels used included ‘my diabetes’, ‘injections’, ‘eye test’, ‘virtual clinic’, ‘the doctor’, ‘nurse’, ‘photographer’, and so forth. A simple count of the number of times that a specific speaker could be linked to a code, and the number of times that one code was associated with another in the same turn, was subsequently used as the basis for the construction of a 20 × 20 square matrix. The matrix was then integrated into social network software (using *Pajek* [23]) to generate a graphical representation of the discussion. Within the graph, node size reflects the number of turns that an individual speaker took during the meeting, or the number of times that an issue was referred to. The thickness of the links between nodes (the arcs) reflects the number of times that any one code was associated with another in the responses of participants. Because the diagram was generated using a Fruchterman–Reingold projection, distances between nodes are suggestive of the closeness (or otherwise) of the links between them (unfortunately, overlapping P2–P4 nodes, had to be separated manually to enhance clarity). Given large variations in the node and arc size, the counts were scaled using a square root transformation. ANX anxiety, AI artificial intelligence for detecting change in retina, DIABETES My diabetes, DRIVE Car Driving, THE DOC Consultant; FEEDBAK Information and results for ‘me’, FOTOG Photographer, INJECT Injections, LAMPEX Slit-lamp exam with doctor, OPTOS Optos imager, VIRT CLINIC Virtual Consultation, WAIT Waiting during routine visits. Participants are labelled ‘Pn.’.

**Fig. 2:**
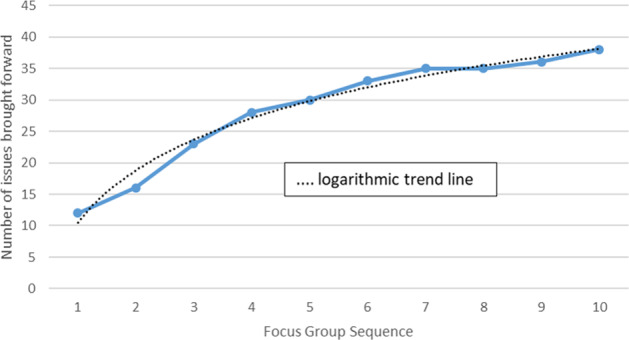
Accumulation of ‘issues’ in 10 consecutive focus groups.

Transcribed data were explored using different forms of content analysis [16, 17]. One strategy is outlined in Fig. 1. Another strategy involved simple counts of topics brought forward by FG participants. Thirty-eight such topics were identified, including those planned by the moderator (the average for the groups as a whole was 15 per meeting). The plot in Fig. 2 shows the number of ‘new’ or additional topics that arose with each meeting. The levelling-off of the accumulation curve is suggestive of a pending data saturation point [18–20]. The curve reflects a characteristic pattern of data collection in qualitative studies, and its shape suggests that, despite the relatively small number of participants, recruitment of further groups and participants would most likely have yielded only few additional issues.

Ethical approval for this study was obtained (17/NI/0124) and the study was conducted following the ethical principles of the Declaration of Helsinki. EMERALD was funded by the Health Technology Assessment of the National Institute for Health Research (HTA-NIHR 15/42/08).

## Results

### The patient point-of-view

Topics of concern emerged in different strengths both within and across FG meetings. Using content analysis and coding Fig. 1 offers a representation of the proceedings in just one FG; it takes the form of an issue web, and it is offered as a summary of conversational proceedings. It shows, for example, that some speakers took more turns than others (P5—smaller red box—spoke least; P1—larger yellow box—the most). It also shows that the major concern of these participants was the ‘feedback’ (large pink box toward the centre of the diagram), i.e. getting information on their eye condition— usually at the time of a clinic visit. It shows participants introduced topics that were not included in the moderator’s script. Examples include references to ‘my diabetes’, driving, and the primary care (general practitioner) service. More importantly, it demonstrates differences in attitude to the acceptance of a virtual consultation (dotted lines between nodes signal negative associations), and the use of fundus imaging equipment (specifically an Optos^TM^ imaging instrument). Thus, participants 2–4 openly disliked the idea of a virtual, or even a grader-led clinic. Finally, we can see that participants frequently cross-reference issues (line thickness indicates a strength of association between one topic and another); indeed, they rarely talked in terms of single issues or themes. For the sake of clarity, however, the presentation of findings that follows is focussed on views relating to the provision of a patient-care pathway in which a consultation with a medical professional is absent. Such views are best summed up via an analysis of three topics, namely (a) the distribution of expertise within the clinic; (b) interaction with clinic professionals and flow of information from professionals to patients; and (c) The needs of people with diabetes. Reactions of clinic professionals to those issues are also presented.

### The distribution of expertise

In the FG meetings issues of expertise, skill, and knowledge were prominent. Participants valued expertise, and in the perceived hierarchy of expertise it was the ophthalmologist that was seen as what one participant in FG6 called, ‘the main one’.

According to FG participants, meeting the ophthalmologist during a clinic visit was above all, ‘reassuring’. Thus, one of the male FG3 participants claimed that reassurance flowed from the fact that “there’s questions that you ask that probably only [the ophthalmologist] could answer”. (A view independently supported by one of the ophthalmic graders at their FG discussion.) Whilst a female participant (FG2) stated that meeting the ophthalmologist offered “a great reassurance … you know you’re going to be looked at”.

Participants recognised other clinic personnel had skills, but such skills had to be underpinned by the expertise of the clinician to carry any weight. Thus, a member of FG4 recognised the photographer (referred to as the “guy on the machine”) was, “skilled at what he does”, but added that his work was always checked by the ophthalmologist. When the issue of skill was raised in FG6, one of the respondents agreed that clinic professionals were “very” skilled, but the skill was “in handling people”. Later in the same group the moderator asked, “Would you imagine the photographer would have diagnostic skills?” The answer was brief, but clear. “No, no”. In FG8 participants suggested they would be happy to get ‘results’ of investigations from the photographer only as long as it was confirmed by the ophthalmologist. Thus, one female speaking of scan results stated, “I would just like it confirmed. If [the photographer] want to tell us, that’s fine, but as long as [the ophthalmologist] has seen everything.” When faced with the same question a member of FG3 stated; “There’s things that can’t be picked up in the photographs but which would be picked up if every patient actually saw the ophthalmologist”. Whilst another member of FG3 suggested that even if the photographer can detect something awry, “well that’s all they’re doing, they’re just seeing the difference, they don’t know why”. A member of FG5 argued that images alone were never sufficient to detect change in the retina and that, “for peace of mind of the patient, they should be seen by the ophthalmologist and get the slit lamp and all the rest of it”. A member of FG10 even pointed to the existence of tacit knowledge in the detection of fundus damage when he stated that by using slit-lamp biomicroscopy the ophthalmologist was likely to see, “Something that the scan hasn’t caught”.

It is clear, then, that patients participating in the FGs recognised a variety of expertise within the clinic combined with an unequal distribution of skill and knowledge among professionals. Nursing staff and photographers were respected for what they did, and the way they did it, but, in the view of patients, they were not sufficiently trained to diagnose or deal with the problems patients faced. Put simply, they were “not as good as the ophthalmologist” (Female, FG 8).

### Interaction with clinic professionals and the flow of information

Issues about information exchange and face-to-face interactions with clinic professionals overlapped with issues about skill and expertise. During FG proceedings, the moderator asked patients whether they talked to the nurse and/or the photographer. As far as nursing staff were concerned, the response of one of the men in FG10 sums up the general position: “There’s no clinical discussion, as such. It’s just ‘how you are today? Left eye, right eye, do you have your glasses with you?” Participants in most of the FGs reported that verbal interactions with the nursing staff were just ‘chat’—e.g. “just about the weather” (FG8).

Nurse–patient interactions were reported as being brief, business-like, and involving a minimal amount of information exchange. Some participants even spoke of a refusal or inability on behalf of nurses to give patients any information. ‘John’ (FG9) stated, “if I ask a question, the nurse will say to me… She’ll say to me, ‘right, I’m unable to answer that question, John, but you’ll be seeing the doctor and he will put you right’”.

Similar patterns of interaction were reported in relation to the meeting with photographers, though with the latter there were some additional complications; namely that patients had little idea about the skills, credentials, or occupational status of the individuals who scanned their eyes. Thus, when the moderator asked members of FG6 whether they ever discussed their condition with the photographers one participant asked, “What photographers do we meet?” On further clarification (from another participant) she then referred to “The lady who runs the scan machine”. Getting information from the photographer was regarded as insufficient, for he or she is after all, “Just the photographer” (FG2).

For patients, asking questions and getting informed answers (‘there and then’) constituted the high point of a routine clinic visit. Indeed, the capacity for getting ‘feedback’, and obtaining ‘information’ was regarded as a central function of patient–doctor interaction, as shown Fig. 1. It was the potential loss of this capacity in any service re-organization that primarily concerned patients. Not getting information—on the day of a clinic visit—offers the worst of all possible worlds; a world set up to generate anxiety and worry. As a member of FG6 stated, “I think that’s the problem, it’s waiting for [the results of a scan]… Like the sword of Damocles hanging over me”. Indeed, leaving the clinic with “an answer”, “that’s the most important thing” (Male, FG9).

### Treating people with diseases

Whenever they reflected on the nature of their eye condition patients frequently referred to ‘worry’, ‘anxiety’, and need for ‘reassurance’. For the participants in FG2, for example, worry, anxiety, fear, nervousness, and the overwhelming need to be reassured about the status of their condition dominated the FG discussion. Even short delays in getting a clinic appointment could be critical. “You’re sitting at home [waiting], and you are terrified you are losing your sight” (Female FG2). Commenting on his clinic’s appointment system, a member of FG3 stated, “I would say that we all worry about our sight being lost”. Whilst in reaction to the first of the virtual clinic vignettes (and the suggestion that the result of eye scans be sent by post) a member of FG8 stated “you’re worrying, you’re anxious. And the thing is, with diabetes, being anxious and stressed doesn’t do it any good”. Commenting on the same vignette a woman in FG6 stated, “That means [the patient’s] got an undefined period of time of worry, probably acute worry”. A second participant in FG10 was quite clear about the wider implications of adopting virtual clinics; “On a practical level, I don’t see a problem with it. On an ideological level, as good practice, it’s not good”.

The aforementioned reference to diabetes serves to underline another important characteristic of this patient group—that diabetes is central to their everyday identity. Diabetes seemingly dominated daily routine and was not a separate feature from the eye conditions. Consequently, many participants argued that “Holistically is the only way to deal with diabetes. If you miss one part of it then, it’ll kill you, that’s the bit that will get you. It’s a killer disease” (Male FG10). Whilst a member of FG4 compared his treatment in two different clinics; suggesting one was clearly preferable to the other in its approach to his health because it adopted what he called a ‘holistic’ approach.

It was for such reasons that provision of a virtual clinical service was variously described as ‘a backward step’, ‘retrograde’, leaving patients ‘isolated’ and ‘cast adrift’, without an opportunity to ‘have a talk [with the ophthalmologist], and get any problems in…’

### Viewpoints of clinic professionals

For photographers, graders, and ophthalmologists the provision of virtual clinics was viewed, for the most part, as a means of organising scarce resources to meet growing demand. In that frame, reservations voiced by patients were ones that supposedly needed to be corrected through ‘patient education’; pointing out, for example, that most patients are ‘low risk’ and have no routine need to see an ophthalmologist. Some ophthalmologists suggested patients were merely ‘resistant’ to change and would soon ‘get used’ to a virtual service. Others proposed various ad hoc responses to the issues of anxiety and worry (e.g. providing patients with their office phone number). Graders and photographers also held to such views, but also expressed reluctance to get involved with detailed discussion with patients about their eye conditions. Providing factual information might be acceptable, but discussing implications (e.g. for treatment) was not.

## Discussion

EMERALD explored patient and health professional’s views on a new care pathway that would not include routine face-to-face examination by the ophthalmologist. Most patients felt that restricting face-to-face clinical interactions would be a negative step; mainly because patients valued a consultation with a medically trained professional from which they could obtain detailed information (‘results’ of investigations), expert opinion, and reassurance.

Professionals who worked in clinics, however, tended to view the adoption of a grader-led clinic (“virtual” or “live”) mainly in the context of a need to rationalise scarce resources; to focus on ‘high risk’ rather than ‘low-risk’ patients. Photographers and consultants argued patient concerns could be best managed via programmes of patient education. In other respects their views echoed those in the available literature, wherein the emphasis is predominantly on technical matters of diagnostic accuracy (using images alone) [21, 22]. Yet the adoption of a grader-led clinic holds important implications for doctor–patient communication, the management of information and anxiety, and the re-distribution of professional skills. In the absence of face-to-face clinics, immediate feed-back to patients from graders, and periodic, even if at less frequent intervals, evaluations by ophthalmologists would be more acceptable to patients and should be considered prior to the introduction of this new care pathway. Patient education regarding professional identity, training and performance of ophthalmic graders would be also essential.

One advantage of this study was a capacity to explore patient views in detail rather than as straightforward responses to structured pre-set questions. The study also drew upon patient views from widely different areas of the UK. Unfortunately, the response rate to FG participation was low—though as previously indicated, there are strong grounds for considering the coverage of opinions robust.

Currently, virtual clinics are used for managing of other chronic eye conditions, such as age-related macular degeneration and glaucoma, besides diabetic retinopathy. Consequently, the results of EMERALD may be useful and applicable to the management of other eye diseases.

## Summary

### What was known before

- There was a lack of information before about the acceptance by patients of allied non-medical staff-led clinics.

### What this study adds

- Through focus group discussions EMERALD found out patients prefer face-to-face examinations done by an ophthalmologist. In their absence, they would accept the new surveillance pathway (ophthalmic grader’s pathway) provided they get immediate feedback and still see the ophthalmologist from time to time.

## Data Availability

All data generated or analysed during this study are included in this published article.
